# Gold Alloy

**Published:** 1883-02

**Authors:** 


Gold Alloy.—Dr. W. H. Dorrance prepares gold solder after this manner: take of pure silver one part, of zinc two parts and of copper three parts. Melt the silver and copper together and add the zinc slowly, in small pieces, allowing all the fumes to pass off. If, after thorough mixing, this is thrown into water, it will separate into small pellets. With these reduce the gold to the proper degree of fineness. If pure gold is used, the number of parts out of twenty-four which are taken will express the fineness of the solder in carats. Twenty parts of gold will make 20-k. solder; sixteen parts, 16-k. solder, etc. If U. S. coin be used, there will be two parts more of alloy, and twenty parts of gold will make but 18-k. solder. After melting it may be run into an ingot, when it will readily roll out into a plate of any desired thickness.

We have used solder made in this manner, and can express our entire satisfaction with it. It bears an excellent color, flows easily and is very strong. We are under obligations to Dr. Dorrance for this formula, as well as for other courtesies.

A Poison for Tubercular Bacteria.—A paper was recently communicated to the Paris Académie des Sciences, by M. de Korab, on the action of helenine on the bacteria of tuberculosis. The facts mentioned deserve notice, although we fear that the hopes suggested are too bright to be realized. The bacilli were cultivated in bovine blood serum, which was daily heated for a week to effectually sterilize it, and was then coagulated by a temperature of 65 degrees C. A guinea pig having been rendered tubercular by inoculation and inhalation, small tubercule masses were taken from it, introduced into ten tubes containing the tubercular serum, and the tubes plugged after some helenine had been poured into three of the tubes. All were kept at a temperature of 37 degrees C. for a week, and at the end of that time, inoculation experiments showed that the organism in the tubes to which the helenine had been added, no longer caused tuberculosis, which was readily produced by the contents of the other tubes.

